# Antagonistic activity of *Ocimum sanctum* L. essential oil on growth and zearalenone production by *Fusarium graminearum* in maize grains

**DOI:** 10.3389/fmicb.2015.00892

**Published:** 2015-09-03

**Authors:** Naveen K. Kalagatur, Venkataramana Mudili, Chandranayaka Siddaiah, Vijai K. Gupta, Gopalan Natarajan, Murali H. Sreepathi, Batra H. Vardhan, Venkata L. R. Putcha

**Affiliations:** ^1^Food Microbiology Division, Defence Food Research LaboratorySiddarthanagar, India; ^2^Toxicology and Immunology Division, DRDO-Bharathiar University Centre for Life SciencesCoimbatore, India; ^3^Department of Biotechnology, University of MysoreMysore, India; ^4^Discipline of Biochemistry, National University of Ireland GalwayGalway, Ireland; ^5^Food Biotechnology Division, Defence Food Research LaboratorySiddarthanagar, India

**Keywords:** *O. sanctum* essential oil, GC–MS, *F. graminearum*, micro-well dilution method, scanning electron microscope, zearalenone, reverse transcriptase qPCR, UHPLC

## Abstract

The present study was aimed to establish the antagonistic effects of *Ocimum sanctum* L. essential oil (OSEO) on growth and zearalenone (ZEA) production of *Fusarium graminearum*. GC–MS chemical profiling of OSEO revealed the existence of 43 compounds and the major compound was found to be eugenol (34.7%). DPPH free radical scavenging activity (IC_50_) of OSEO was determined to be 8.5 μg/mL. Minimum inhibitory concentration and minimum fungicidal concentration of OSEO on *F. graminearum* were recorded as 1250 and 1800 μg/mL, respectively. Scanning electron microscope observations showed significant micro morphological damage in OSEO exposed mycelia and spores compared to untreated control culture. Quantitative UHPLC studies revealed that OSEO negatively effected the production of ZEA; the concentration of toxin production was observed to be insignificant at 1500 μg/mL concentration of OSEO. On other hand ZEA concentration was quantified as 3.23 μg/mL in OSEO untreated control culture. Reverse transcriptase qPCR analysis of ZEA metabolic pathway genes (*PKS4* and *PKS13*) revealed that increase in OSEO concentration (250–1500 μg/mL) significantly downregulated the expression of *PKS4* and *PKS13*. These results were in agreement with the artificially contaminated maize grains as well. In conlusion, the antifungal and antimycotoxic effects of OSEO on *F. graminearum* in the present study reiterated that, the essential oil of *O. sanctum* could be a promising herbal fungicide in food processing industries as well as grain storage centers.

## Introduction

The existence of fungal species and their toxic secondary metabolites viz. mycotoxins in food and feed commodities is a major concern for microbiological safety and food security (Pitt and Hocking, 2009). Fungi and their mycotoxins could cause massive financial loss to global economy, because of their deleterious effects in cereal crops besides humans and farm animals (Rocha et al., 2014). Among the toxigenic and pathogenic fungal species, *Fusarium graminearum* has been given special attention in agriculture commodities due to its ability to grow in diverse climatic conditions, and to produce different mycotoxins including ZEA and type-B trichothecenes (Morgavi et al., 2007; Bernhoft et al., 2012). ZEA also known as F-2 or RAL is a temperature stable, persistent, moderately water soluble, non-steroidal estrogenic mycotoxin (Zinedine et al., 2007). As one of the most prevalent mycotoxins, its presence has frequently been analyzed in agricultural as well as environmental products (Zinedine et al., 2007). In a recent study, Cano-Sancho et al. (2012) and Tomoya et al. (2014) reported that, cereals and food grain samples have been found to be heavily contaminated with ZEA (concentration ranging from 3.1 to 5.9 μg/kg) and at the maximum concentration of 153 μg/kg ZEA was reported in Job’s tears product. In another study, Pleadin et al. (2012) reported a maximum concentration of 5.11 mg/kg ZEA in maize.

Zearalenone binds to estrogen receptors (ERs) ending up in estrogenicity, which occasionally brings hyperestrogenism in livestock and humans, particularly in females (Zinedine et al., 2007). The toxic effects of ZEA as noticed both in the laboratory and household animals include endocrine interruption leading to induction of the expansion of estrogen-sensitive cells and tissues, abnormal feminization of male gonads or reproductive system disorders, skeletal distortion, cancer, weakening of bones, and myelofibrosis (Zinedine et al., 2007). In our recent study Venkataramana et al. (2014) reported the neurotoxic and genotoxic effects of ZEA in human neuronal (SH-SY5Y) cells. Also in another study Richard (2007) reported genotoxic role of ZEA by *in vitro* methods through SOS repair, chromosomal aberration and sister chromatid substitution. Owing the to its estrogenic and carcinogenic effects of ZEA to humans and other farm animals, International Agency for Research on Cancer (IARC) has defined ZEA as a Group 3 carcinogen (IARC, 1999). Due to the above said implications, many regulatory agencies proposed the maximum permissible limits for ZEA in several food matrices. The European Union (EU) has established allowed limits for ZEA in unprocessed cereals as 100 μg/kg excluding maize and the permitted level in unprocessed maize was 350 μg/kg (European Commission, 2007). The Joint FAO/WHO Expert Committee on Food Additives (JECFA) has set up a Provisional Maximum Tolerable Daily Intake (PMTDI) of 0.5 μg/kg body weight (JECFA, 2000).

To date, many synthetic antifungal agents have been licensed for successive control of *F. graminearum* as well as other fungi. Unfortunately, the application of synthetic antifungal agents in agricultural commodities was responsible for a multitude of negative health impacts in livestock and humans and also resulted in upsurge of resistant organisms as well (da Cruz Cabral et al., 2013). Therefore, there is a need to propose proper food grain management practices, including the application of herbal antifungal and mycotoxin controlling agents, thus to reduce the growth of toxigenic *F. graminearum* as well as the production of ZEA in agricultural commodities. In this instance, wide varieties of secondary metabolites produced by plants are offering options for synthetic antifungal agents because of their bio-degrading nature as well as non-toxic to the environment. Essential oils obtained from the plants have always been an excellent source of antioxidant compounds such as polyphenols, flavonoids, etc., which have been believed to be the basis of their antifungal capabilities (da Cruz Cabral et al., 2013). Essential oils are aromatic liquids attained through hydrodistillation from the plant material and they are constituted by a great diversity of compounds confers many advantages, such as possessing unique modes of action like antioxidant, antimicrobial, and anticancerous properties against many infectious pathogens and life-threatening diseases (Raut and Karuppayil, 2014).

*Ocimum sanctum*, also known as the Holy Basil or Tulsi is widely used as a medicinal plant in Ayurveda. The plant has been traditionally used in management of common cold, asthmatic illnesses, skin problems, urogenital infections, digestive, neurological and cardiovascular disorders (Gupta et al., 2002). The chemical constitution of OSEO includes a variety of active compounds, including eugenol, methyl eugenol, methyl chavicol, α-terpineol, germacrene D, β-caryophyllene, camphor, camphene, β-ocimene, β-elemene, linalool, 1,8-cineole etc. (Kothari et al., 2005; Khan et al., 2010; Joshi, 2013). Kumar et al. (2010, 2013) reported the inhibitory activity of OSEO on growth of *Aspergillus flavus* NKDHV8 and *A. flavus* LHPRS7 isolated from raw materials of *Rauvolfia serpentina*, respectively. Sethi et al. (2013) reported strong inhibitory effect of OSEO (MIC of 62.5 μg/mL) against *Rhizoctonia solani* and Khan et al. (2010) reported the antifungal activity of OSEO on *Candida sp.* by the mechanism of disrupting ergosterol biosynthesis and membrane integrity. In contrast, the application of OSEO as the antifungal agent in management of toxigenic *F. graminearum* and ZEA production are still unexplored. Also, no previous study investigated the applicability of OSEO on grain samples.

The objective of the present study is to establish the antagonistic effects of OSEO on growth and ZEA production of *F. graminearum*. OSEO was extracted from aerial parts of *O. sanctum* L. by hydrodistillation method and chemical profile was carried out by GC–MS method. Antioxidant and antifungal activities of OSEO were carried out by DPPH free radical scavenging activity, and micro-well dilution and scanning electron microscope methods, respectively. Effect of OSEO on ZEA production was analyzed by Rt-qPCR and UHPLC methods from broth culture of *F. graminearum*.

## Materials and Methods

### Chemicals and Reagents

All the chemicals (analytical grade) and solvents (HPLC grade) were procured from Merck (Bangalore, India). Plasticware used in the study were obtained from Eppendorf (Hamburg, Germany). ZEA standard was obtained from Sigma-Aldrich (Bangalore, India) and stock solution of ZEA was prepared in acetonitrile and stored at -20°C until use. Sabouraud Dextrose media, peptone and nystatin were purchased from HiMedia (Mumbai, India).

### Plant Material Collection and Essential Oil Extraction

The aerial parts of *O. sanctum* were collected from Mysore, Karnataka state, India. The identification and verification of plant was carried at the Botanical Survey of India (Southern Regional Centre, Coimbatore, India) and the voucher was safeguarded at Food Microbiology Division, Defence Food Research Laboratory (Mysore, India). The collected material was air-dried in the shade at 37°C for 4 weeks and used for analysis. The essential oil was extracted from 250 g of the dried plant materials by hydrodistillation using a Clevenger-type device in accordance with the technique approved by the European Pharmacopoeia (Council of Europe, 1997). The oil gathered was separated and dried over anhydrous sodium sulfate to remove water and further stored at 4°C in the dark until use.

### GC–MS Analysis

The GC–MS analysis of OSEO was carried out on PerkinElmer Clarus 600 C (Waltham, MA, USA) analytical system equipped with DB-5MS (30 m × 0.25 mm; 0.25 μm film thickness) merged silica capillary column and attached to flame-ionization detector (FID). OSEO was diluted in acetone (10 μL/mL) and 1 μL solution was injected in a split-mode (1:30). Working conditions were as follows: carrier gas was He (1 mL/min); temperatures were set as follows: injector at 250°C and sensor at 280°C, whilst the column temperature was linearly scheduled 40–280°C at 4°C/min. Mass spectra were documented in EI mode (70 eV) with a range of m/z 40–450. Turbo Mass software application was adapted to operate and acquire data from the GC–MS. The detection of the individual components was achieved by comparison of their mass spectra (MS) with those from accessible libraries (NIST/Wiley) and experimentally determined retention indices (RI) with data from the literature (Adams, 2007). The percentage of the constitution of the individual components was derived out from the peak areas, devoid of correction factors.

### Determination of Antioxidant Activity by DPPH^∗^ Assay

2,2-diphenyl-1-picrylhydrazyl (DPPH) is a constant free radical which responds with compounds, which tend to be able to donate a hydrogen atom (HAT). Therefore, the hydrogen donating capability of OSEO to DPPH free radical (DPPH^∗^) was determined from the change in absorbance at 517 nm in accordance with the method of Ojeda-Sana et al. (2013) with slight modifications. For the radical scavenging measurements, 1 mL of methanol, 1 mL of 0.1 M acetate buffer (pH 5.5), 0.5 mL of a 250 μM methanolic solution of DPPH^∗^ were blended with various concentrations of OSEO (2–18.5 μg/mL). The control was made from the reaction blend without test sample and quercetin was considered as the reference sample. Subsequently the absorbance was determined using BioSpectrometer (Eppendorf, Germany) after 30 min of incubation at 37°C in the dark. The result was stated as IC_50_ (μg/mL), which means that the quantity of sample required to reduce the absorbance of DPPH by 50%. DPPH^∗^ scavenging activity was calculated by the formula,

$$\%\;Scavenging\,\;activity=\frac{A(control)-A\left(test\right)}{A\left(control\right)}\times 100$$

Where, A (control) was the absorbance of the control (without test sample) and A (test) was the absorbance of the test sample.

### Antifungal Activity

Zearalenone producing *F. graminearum* (MTCC, 1893) was obtained from the Microbial Type Culture Collection and Gene Bank, Chandigarh, India (MTCC). *F. graminearum* was grown on SDA for 7 days at 28°C and spores were recovered using peptone water containing 0.001% Tween 80 with soft scrape. The spore number was determined using haemocytometer and spore suspension was adjusted to 1 × 10^6^ per mL. Antifungal activity of OSEO was determined by micro-well dilution method and further it was validated quantitatively by scanning electron microscope observation.

#### Micro-Well Dilution Method

Minimum inhibitory (MIC) and minimum fungicidal (MFC) concentrations were determined by micro-well dilution technique in 96 well microtitre plates with minor modifications (Clinical and Laboratory Standards Institute [CLSI], 2008; Vieira et al., 2014). SDB was used as the media in the well to which 0.001% Tween 80, different concentrations of the OSEO and a volume of 10 μL spore suspension (1 × 10^6^ spores/mL) were added, and the final volume was 100 μL per well. The wells without OSEO were referred as control and microplates were incubated for 3 days at 28°C. The minimum concentration without detectable growth was determined as the minimal concentration which absolutely inhibited fungal growth (MIC). A volume of 10 μL from each well was inoculated into the SDA plates and incubated at 28°C for 3 days, and the minimum concentration with no detectable growth was determined as the MFC, specifying 99.5% killing of the original inoculum in comparison to nystatin, used as a positive control.

#### Effect of OSEO on Spore Germination

Effect of OSEO on spore germination of *F. graminearum* was analyzed by the method of Rana et al. (1997) with minor modifications. A volume of 10 μL fungal spore suspension (1 × 10^6^ spores per mL) was inoculated on SDA slides containing different concentrations of OSEO (100–1800 μg/mL) and incubated at 28°C for 24 h. SDA slide alone with fungal spores and without OESO were considered as control. Following incubation period, each slide was fixed with lactophenol-cotton blue and observed under microscope (Leica DM1000 LED, Leica Microsystems, Wetzlar, Germany) for the spore germination. About 200 spores were examined from each slide and percentage of spore germination was calculated by the formula,

% Spore⁢ ger⁢mination⁢=ST/SC×100

Where, SC was number of spores germinated in control and ST was number of spores germinated in test.

#### Scanning Electron Microscopic Observation

To know the effects of OSEO on mycelial and spore structure of *F. graminearum*, scanning electron microscope (SEM) analysis was performed according to the method of Yamamoto-Ribeiro et al. (2013) with minor modifications. A seven day old mycelia was inoculated aseptically into SDA dishes that contained 1250 and 1800 μg/mL concentration of OSEO and the dishes were incubated at 28°C for 7 days. The control was performed in SDA medium without OSEO. After the incubation period, mycelia disk of 1 cm^2^ was collected and rinsed in phosphate-buffer saline (0.01 M) and fixed with 2.5% glutaraldehyde in 0.1 M sodium cacodylate buffer, pH 6.5 and dehydrated with gradient ethanol (20, 40, 70, 90, and 100%, keeping the mycelia for a longer duration in 100%). The sample was pasted on dual side glue carbon tape and it was fixed to the surface of aluminum stubs. Further, the stubs were exposed toward critical-point dry out in CO_2_ and sputter-coated with gold to increase its conductivity. The morphological quality of the mycelia was observed under scanning electron microscope (FEI, USA) at 20.0 KV in environmental mode.

### Determination of Antimycotoxic Activity of OSEO in Liquid Cultures

Different concentrations of OSEO including, 250, 500, 1000, 1500, and 2000 μg/mL were added to each 250 mL Erlenmeyer flask individually that contained 100 mL of SDB. A volume of 10 μL fungal suspension (1 × 10^6^ spores/mL) of 7-day-old culture was inoculated in these flasks and the flask without OSEO was considered as untreated control. Inoculated flasks were incubated under shaking condition (140–160 rpm) at 28°C for 14 days.

#### Determination of Mycelial Biomass

Following the incubation period the culture media was separated from fungal biomass by filtering through Whatman no. 1 paper and the broth was used for ZEA determination. The fungal mycelia was washed twice with sterile distilled water and 10 mg of mycelia was employed for RNA extraction, and leftover mycelia was dried out on pre-weighed Whatman no. 1 filter paper at 60°C for 24 h and weighed (Denver instruments, India).

#### UHPLC Determination and Quantification of Zearalenone

Detection and quantification of ZEA were carried out as reported by the method of Ibáñez-Vea et al. (2011) with slight modifications. An equivalent quantity of acetonitrile was added to each culture broth and blended thoroughly for 30 min. Subsequently, the sample was centrifuged for 12 min at 6000 rpm and 15 mL of the supernatant was transferred through an immunoaffinity column of ZEA (Vicam, USA), pre-conditioned under 10 mL of phosphate buffer saline (PBS). After the sample had passed away, the column was washed with 5 mL of PBS and 10 mL of distilled water. Finally, the column was dried out with air and ZEA was eluted with 5 mL of acetonitrile, after retaining in contact between acetonitrile and column antibodies for 5 min. The extract was dried out completely over water bath at 60°C and final residue was redissolved in 1 mL of acetonitrile and filtered through 0.22 μm syringe filter. The filtrate was used for the UHPLC determination and quantification of ZEA.

The Nexera UHPLC system (Shimadzu, Kyoto, Japan) attached with the column C18, 5 μm, 250 × 4.6 mm (Phenomenex, USA) was employed for detection and quantification of ZEA carried out in reverse-phase with a fluorescence detector that was adjusted at 334 nm excitation and 450 nm emission. The mobile phase was acetonitrile-water (50:50 v/v) with a flow rate of 1 mL/min. A standard ZEA (100 ng–500 μg/mL) was used to construct a five-point calibration curve of peak areas versus concentration. The injection volume was 25 μL for both the standard solution and sample extracts. The sensing limitation of the technique was 100 ng/mL.

#### Reverse Transcriptase qPCR (Rt-qPCR) Analysis of Zearalenone Metabolic Synthesis Genes

Reverse transcriptase qPCR (Rt-qPCR) evaluation was done to analyze the impact of OSEO on gene expression of *PKS4* and *PKS13*, which are involved in ZEA biosynthesis in *F. graminearum* (Kim et al., 2005; Gaffoor and Trail, 2006) and *GAPDH* was used as endogenous reference gene. Primers were designed against target genes using the GeneRunner software version 5.0.47 Beta (**Table 1**) and synthesized primer sequences were obtained from Sigma-Aldrich (Bangalore, India). Total RNA was extracted using RNA easy plant Mini kit following manufacturer’s guidelines (Qiagen, USA). Briefly, mycelia were flash-frozen in liquid nitrogen and grounded into a fine powder with a porcelain mortar. The total RNA was extracted and quantified by NanoDrop 8000 Spectrophotometer (Thermo Scientific, USA). The analysis of Rt-qPCR was carried out in the Light cycler 480 (Roche, USA) using iScript One-Step RT-PCR Kit with SYBR Green (BIO-RAD). In concise, 50 μL volume of reaction mixture consists of 25 μL of 2X SYBR Green RT-PCR reaction mix, 1 μL of iScript reverse transcriptase for one-step RT-PCR, 1 μL of primer (450 nM), 1 μL of template RNA (100 ng) and 22 μL of nuclease-free water (PCR grade). The thermal conditions for reaction include 10 min of cDNA synthesis at 50°C for 1 cycle, 5 min of polymerase activation at 95°C and followed by 35 cycles of PCR at 95°C for 10 s, 60°C for 30 s for combined annealing and extension. For each and every PCR product, an individual narrow peak was attained through melting curve analysis of the distinct temperatures. The relative quantification levels of expression had been quantified making use of second derivative maximum analysis with the determination of the crossing points for every single transcript. Crossing point values for each gene were normalized to the particular crossing point values for the reference gene *GAPDH*. Data are shown as normalized ratios of genes together with standard error by means of Roche Applied Science E-Method (Tellman and Olivier, 2006).

**Table 1:** Primers used for reverse transcriptase qPCR (Rt-qPCR) analysis of zearalenone production.

Gene targeting	Primer sequence (5′ to 3′)	Tm (°C)
*GAPDH*-F	TATCACGTCTGCCACGAT	56
*GAPDH*-R	CATGTAGGCCTGTGATGA	
*PKS13*-F	TTACCCGCCTCGTTAAAG	56
*PKS1*3-R	AGCTGGCTAAGCGAGGCA	
*PKS4*-F	ATCGGTCATCTTGAGGCT	58
*PKS4*-R	CCGTAGAGAATGCTTTGT	

### Antimycotoxic Property Evaluation of OSEO Onto Maize

Antimycotoxic efficacy of OSEO was assessed directly onto *F. graminearum* inoculated maize grains. The seeds were sterilized by autoclave and dried in hot-air oven at 60°C for 2 h. One 100 g of sterilized maize grains was treated with various concentrations (250, 500, 1000, 1500, and 2000 μg/g) of OSEO in 500 mL conical flask and a volume of 10 μL fungal spore suspension (1 × 10^6^ spores/mL) of 7-day-old culture was inoculated in each conical flask. The grains not treated with OSEO were referred as control and incubated for a period of 14 days at 28°C in a dark condition. Following the incubation period, total RNA was extracted from fungal mycelia and Rt-qPCR evaluation for *PKS4* and *PKS13* genes were carried out as mentioned in “Reverse Transcriptase qPCR (Rt-qPCR) Analysis of Zearalenone Metabolic Synthesis Genes.” Further, maize grains were ground into fine powder and dissolved in 500 mL of acetonitrile and centrifuged at 6000 rpm for 30 min. A volume of 15 mL supernatant was transferred through ZEA specific immunoaffinity column and ZEA was quantitatively determined by UHPLC as mentioned in “UHPLC Determination and Quantification of Zearalenone.”

### Statistical Analysis

All experiments were carried out in six independent replicates and the results were statistically evaluated applying one-way ANOVA for multiple comparisons adapted by the Tukey’s test. Differences were considered statistically significant at a value of *p* < 0.05. Statistical program GraphPad Prism 5.0 (GraphPad Software, Inc., USA) was used to draw graphs.

## Results and Discussion

### Chemical Composition

Based on the dry weight calculation, the yield of OSEO was determined as 1.79% (w/w). Chemical profile of OSEO was revealed by GC-MS analysis and a total of 43 compounds were identified accounting to 98.03% of the total weight (**Table 2**). Among the identified compounds, eugenol (34.7%) was the major compound together with other active compounds with varied concentrations such as thymol (2.98%), linalool (4.94%), β-phellandrene (4.71%), α-phellandrene (3.79%), limonene (3.73%), germacrene D (2.89%), β-pinene (2.59%) and *trans*-pinocarveol (2.37%). Kothari et al. (2005) that methyl eugenol was the major compound (72.5, 75.3, 83.7, and 65.2%) and β-caryophyllene was the second most dominant compound (5.5, 6.4, 2.7, and 12.0%) in the essential oils extracted from whole herb, leaf, stem, and inflorescence of the *O. sanctum* L. from southern India. On the other hand, Kumar et al. (2010) with their study on chemical composition of OSEO from North Indian plants reported that eugenol (61.30%) was a major compound. Recently Verma et al. (2013) explored the chemical profile diversity of OSEO from Indian flora and distinguished the profile into three chemotypes i.e., eugenol, methyl eugenol, and caryophyllene. From the present study, it can be deduced that the chemotype of the *O. sanctum* L. plant in the present study was eugenol chemotype. Our results were also supported by the available literature with respect to chemical composition as there was no new compound observed. The significant differences in the concentration of the determined compounds in comparison with existing reports could be explained by the variation in climatic conditions and harvesting period, luminosity as well as oil extraction method (Kothari et al., 2004; Gobbo-Neto and Lopes, 2007; Verma et al., 2013).

**Table 2:** Chemical composition of the essential oil of *Ocimum sanctum*.

S. No	Compound^a^	RI^b^	RI^c^	Quantity (%)
1	α-Thujene	926	924	0.18
2	α-Pinene	933	932	1.52
3	Camphene	949	946	1.71
4	Sabinene	971	969	0.36
5	β-Pinene	977	974	2.59
6	Myrcene	989	988	1.66
7	α-Phellandrene	1003	1002	3.79
8	δ-3-Carene	1010	1008	0.39
9	α-Terpinene	1017	1014	0.83
10	*p*-Cymene	1021	1020	0.41
11	o-Ocimene	1023	1022	0.24
12	Limonene	1024	1024	3.73
13	β-Phellandrene	1027	1025	4.71
14	(E)-β-Ocimene	1047	1044	1.30
15	γ-Terpinene	1057	1054	1.27
16	Linalool	1099	1095	4.94
17	n-Nonanal	1105	1100	0.61
18	*trans*-Pinocarveol	1140	1139	2.37
19	Camphor	1143	1141	1.70
20	Isoborneol	1156	1155	1.29
21	Pinocarvone	1163	1164	3.86
22	Borneol	1166	1165	1.81
23	α-Terpineol	1187	1186	0.29
24	Linalyl acetate	1256	1254	1.29
25	Bornyl acetate	1288	1287	1.37
26	Thymol	1291	1289	2.98
27	δ-Elemene	1336	1335	0.53
28	α-Cubebene	1349	1345	0.68
29	Eugenol	1360	1356	34.7
30	α-Ylangene	1372	1373	1.20
31	α-Copaene	1377	1374	0.22
32	β-Elemene	1390	1389	0.17
33	β-Caryophyllene	1420	1417	1.29
34	α-Humulene	1455	1452	0.17
35	γ-Muurolene	1480	1478	0.19
36	Germacrene D	1486	1484	2.89
37	β-Selinene	1491	1489	1.23
38	α-Muurolene	1506	1500	1.71
39	γ-Cadinene	1517	1513	1.28
40	δ-Cadinene	1525	1522	0.52
41	Elemol	1551	1548	0.92
42	Cubenol	1640	1639	1.74
43	Bulnesol	1673	1670	1.39
**Total**				98.03%

*^a^Compounds are indexed in order of their elution.*

*^b^Retention indices of compounds determined based on n-alkanes (C-9–C-24) on DB-5MS column.*

*^c^Retention indices of compounds on DB-5 column in accordance with Adams (2007).*

### Antioxidant Activity

**FIGURE 1:**
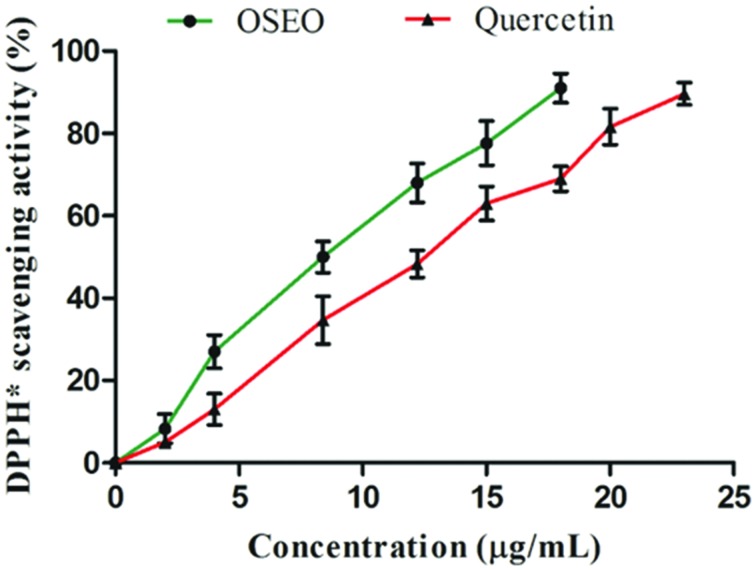
**Dose-dependent antioxidant activities of OSEO and quercetin were determined by DPPH^∗^ assay.** Data were analyzed applying a one-way analysis of variance (ANOVA) followed by the Tukey’s test (*n* = 6) and *p*-value <0.05.

*In vitro* antioxidant potential of OSEO was carried out by DPPH free radical scavenging assay based on single electron (SET) and a HAT transfer reactions (Huang et al., 2005). In the present study, free radical scavenging activity of the OSEO was directly proportional to the OSEO concentration (**Figure 1**) and antioxidant potential of the OSEO showed a higher free radical scavenging activity compared with that of reference antioxidant quercetin. The value for 50% scavenging activity (IC_50_) of OSEO was 8.5 μg/mL whereas quercetin was 12 μg/mL. Joshi (2013) reported antioxidant activity (IC_50_) of OSEO by DPPH^∗^ assay as 219.16 ± 1.01 μg/mL. Interestingly, in the present study reported antioxidant potential of OSEO was quite high compared to earlier report of Joshi (2013). The chemical profile of OSEO in the present study revealed that, in addition to eugenol many other active compounds such as α-thujene, α-pinene, camphene, camphor, limonene, α-phellandrene, β-phellandrene, linalool, linalyl acetate, *trans*-pinocarveol, pinocarvone, germacrene D, β-caryophyllene and thymol were reported, which were absent in the report of Joshi (2013). This might be the reason for enhanced antioxidant activity of OSEO in the present study. Trevisan et al. (2006) also reported DPPH^∗^ scavenging activity (IC_50_) of OSEO as 0.26 μL/mL. The antioxidant potential of essential oil is mainly associated with the presence of phenolic compounds participating in SET and/or HAT reactions. This may be due to the extent of structural conjugation and the presence of electron-donating and electron-accepting substituents on the ring structure of phenolic compounds (Ložienė et al., 2007). Earlier *in vitro* studies proved that oxidant stressors enhanced the mycotoxin biosynthesis and the use of plant derived antioxidant supplements are effective in down regulating the production of mycotoxin (da Cruz Cabral et al., 2013). Similar results were obtained by Kumar et al. (2010) in the case of effect of OSEO in controlling growth and aflatoxin B1 in *Aspergillus flavus*. The observed high antioxidant activity of OSEO indicated its applicability in affecting the mycotoxin production by fungi.

### Antifungal Activity

**FIGURE 2:**
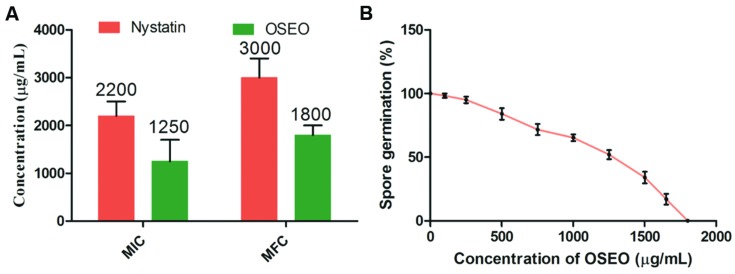
**(A)** Minimum inhibitory concentration (MIC) and minimum fungicidal concentration (MFC) of OSEO and nystatin were determined by micro-well dilution method. **(B)** Effect of different concentrations of OSEO on spore germination of *Fusarium graminearum*. Data were analyzed applying a one-way ANOVA followed by the Tukey’s test (*n* = 6) and *p*-value <0.05.

The antifungal property of OSEO although was highly exploited in previous studies, no reports ever existed on antifungal activity on *F. graminearum* and ZEA production. In the present study OSEO showed MIC and MFC at a range of 1250 and 1800 μg/mL, respectively against *F. graminearum*. This was significantly higher (*p* < 0.05) when compared to reference drug nystatin that showed MIC and MFC activity at 2200 and 3000 μg/mL, respectively (**Figure 2A**). Alternatively, we also carried out fungal spore germination susceptibility assay to support the antifungal activity of OSEO. The results of the spore germination studies reveled that studied concentrations of OSEO showed significant control in *F. graminearum* spore germination on SDA compared to the untreated cultures (**Figure 2B**). A decrease in spore germination was observed with increasing the concentration of OSEO and 100% inhibition of spore germination was observed at 1800 μg of OSEO.

The variability and diversity of the chemical composition and the wide spectrum antimicrobial activities of OSEO, as supported by the present study and previous literature, could be indicative for further studies to be undertaken to delineate the specific compound in OSEO, which might be a major responsible factor for the aforementioned activities. In Campaniello et al. (2010) reported the role of eugenol in antifungal activity on *Penicillium*, *Aspergillus*, and *Fusarium* species. These findings further supported by Dalleau et al. (2008) and da Cruz Cabral et al. (2013), who reported that, phenolic compounds have an ability to disrupt the lipid bilayer of cell membrane and mitochondria, thus to cross the cell membrane and interacting with the enzymes and proteins of the membrane leads to functional alterations in the cell as well as mitochondrial dysfunction this in turn lead to apoptotic cell death.

**FIGURE 3:**
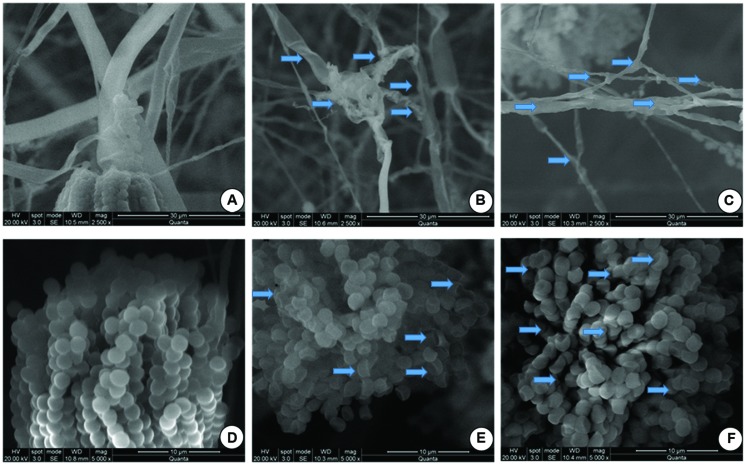
**Scanning electron microphotographs of hyphae and spores of *F. graminearum* grown on SDA with or without OSEO during 7 days of incubation at 28°C. (A)** Hyphae without OSEO (Control), **(B)** Hyphae with OSEO at a concentration of 1250 μg/mL, **(C)** Hyphae with OSEO at a concentration of 1800 μg/mL **(D)** Spores without OSEO (Control), **(E)** Spores with OSEO at a concentration of 1250 μg/mL, **(F)** Spores with OSEO at a concentration of 1800 μg/mL.

In the present study, effect of OSEO on micro morphology of *F. graminearum* was confirmed and validated by SEM observations at MIC (1250 μg/mL) and MFC (1800 μg/mL). Significant effects of OSEO on micro morphology of *F. graminearum* mycelial structure as well as spore structure were observed. The control hyphae showed healthy morphology with smooth, turgid, homogenous surface without any discernible change (**Figure 3A**). On the other hand, morphology of OSEO treated hyphae underwent noteworthy alterations and showed evident modifications in both apical regions and throughout the length of hyphae. The cell wall displayed an irregular surface appearance with craters and protuberances. In addition, hyphae were severely collapsed and squashed due to lack of cytoplasm and a few small vesicles were observed on the apical surface of the mycelia (**Figures 3B,C**). Spores treated with OSEO at concentrations of MIC and MFC values exhibited wrinkled, disrupted, and dispersed appearance compared to the untreated sample (**Figures 3D–F**). Earlier study by Wang et al. (2010) on effect of eugenol on *Botrytis cinerea* morphology revealed similar damages to the structure of hyphae. Also, Yamamoto-Ribeiro et al. (2013) observed similar morphological aberrations in *Zingiber officinale* essential oil (ZOEO) treated *F. verticillioides* hyphae. When the composition of ZOEO was analyzed, 7.73% of the oil was composed of β-phellandrene, a significant compound (4.71%) observed in OSEO also. These observations suggest that in the present study, the damage may be mediated via the most abundant component of the OSEO viz., eugenol and another significant compound, β-phellandrene.

### Antimycotoxic Activity of OSEO on Liquid Cultures

**FIGURE 4:**
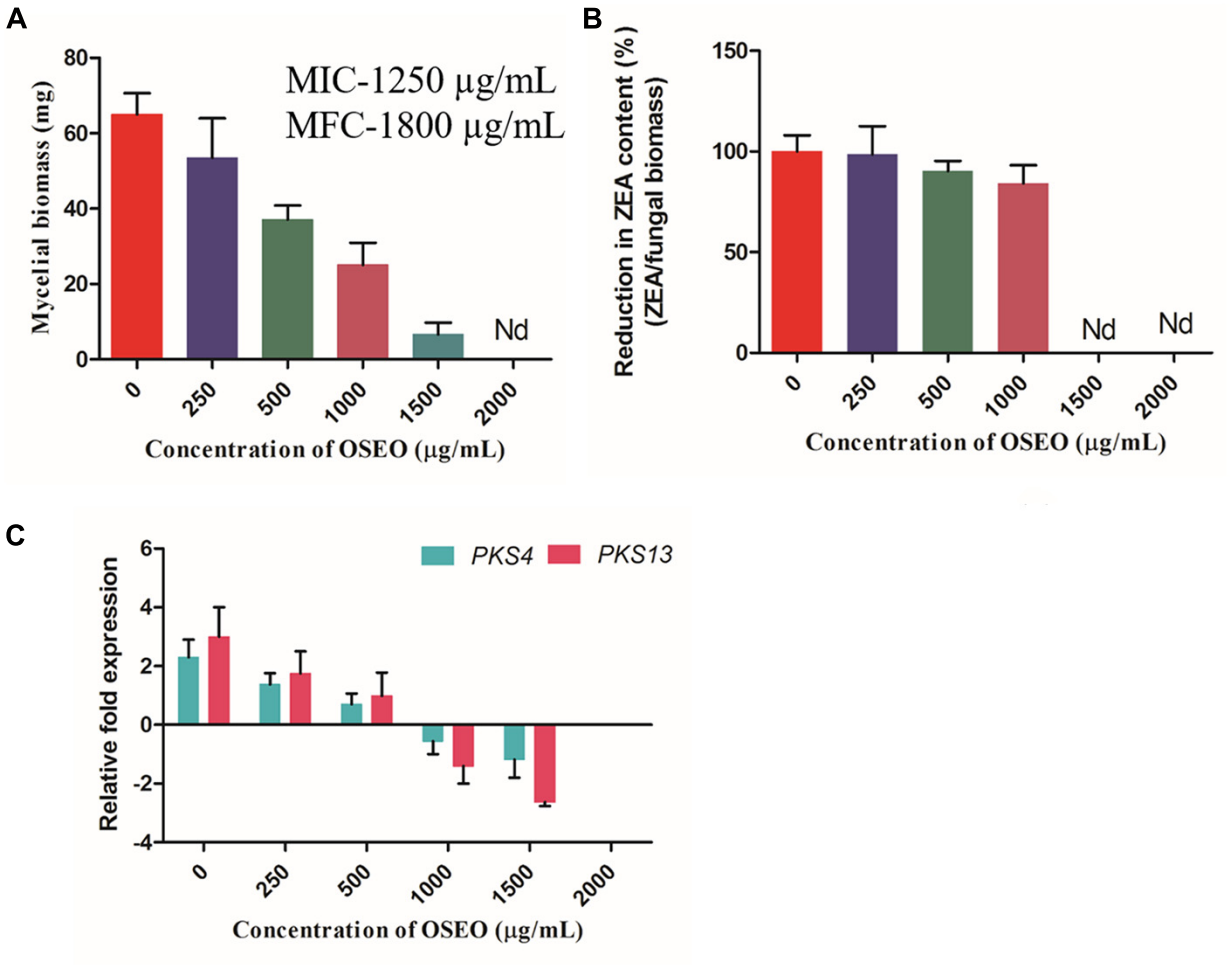
**Effect of OSEO on mycelial dry weight and ZEA toxin production in *F. graminearum*.** The experiment was conducted for 14 days of incubation period at 28°C. **(A)** Effect of various concentrations of OSEO on dry weight of mycelia. **(B)** Correlation analysis between the reductions in fungal biomass versus reduction in ZEA concentration with that of OSEO concentration, results were expressed in percentage of ZEA reduction. **(C)** Effect of various concentrations of OSEO on zearalenone biosynthetic pathway genes expression (*PKS4* and *PKS13*) by reverse transcriptase qPCR (Rt-qPCR). Data were analyzed applying a one-way ANOVA followed by the Tukey’s test (*n* = 6) and *p*-value <0.05. Nd, not detected.

The ultimate aim of the present study was to control the ZEA production by *F. graminearum* from contaminated cereal grains and other food samples intended for consumption. Hence, as an objective of the present study, effects of OSEO on mycotoxin (ZEA) production in liquid cultures was determined. Results of the present study, showed that OSEO has multifaceted efficacy in inhibiting ZEA production in *F. graminearum*. The mycelial biomass, gene expression (*PKS4* and *PKS13*) and ZEA production exhibited a significant declining trend with increasing concentration of OSEO, i.e., reduction of mycelial biomass and metabolic pathway gene expression causing significant reduction in ZEA production (**Figure 4**). Mycelial biomass was reduced in a dose-dependent manner upon the treatment of OSEO compared to untreated control. The dry weight of OSEO untreated control fungal biomass was estimated as 65.0 mg, while OSEO treated samples showed significant decrease in mycelia dry weight as 53.5, 37.0, 25.1, and 6.6 mg in OSEO concentrations of 250, 500, 1000, and 1500 μg/mL, respectively. However, there was no significant growth observed at a concentration of 2000 μg/mL or more of OSEO (**Figure 4A**).

The analysis of the culture broth for ZEA production by quantitative UHPLC analysis showed that, there was a significant decline in the ZEA production as followed by the OSEO concentration. The concentration of ZEA varied with varying in OSEO concentration viz, 2.62 μg/mL in 250 μg/mL, 1.66 μg/mL in 500 μg/mL and 1.05 μg/mL in 1000 μg/mL in comparison with OSEO untreated control (3.23 μg/mL of ZEA). However, at OSEO concentration of 1500 μg/mL or more, no ZEA production was observed. Results of the present study clearly indicated that, fungal growth was reduced significantly upon increasing OSEO concentration and at higher concentrations (2000 μg/mL) no observable fungal growth was recorded. This in turn is directly proportional to the ZEA concentration as analyzed by UHPLC. To know the relation between mycelial biomass and ZEA concertation upon OSEO treatment, correlation analysis was undertaken and these results suggested that, there is significant linearity between the reductions in fungal biomass versus reduction in ZEA concentration (**Figure 4B**) with that of OSEO concentrations used. The amount of toxin produced was normalized by the biomass of mycelium collected and the percentage of ZEA content in OSEO untreated control was considered as 100%. The reduction in ZEA content of OSEO treated samples were expressed as a percentage of ZEA content in untreated control and the percentage of reduction in ZEA content observed as 98.55, 90.29, and 84.18% at OSEO concentrations of 250, 500, and 1000 μg/mL, respectively (**Figure 4B**).

To further assess the mechanism behind this inhibition, we studied the effects of OSEO on regulation of target metabolic pathway genes (*PKS4* and *PKS13*) recorded in terms of mRNA expression and results were expressed as a fold change in normalization with reference control gene *GAPDH*. Results of the Rt-qPCR revealed that, upon treatment of *F. graminearum* with OSEO, *PKS4* and *PKS13* expression levels were downregulated with increase in OSEO concentration compared to the OSEO untreated *F. graminearum* culture. The downregulated gene expression levels of *PKS4* and *PKS13* were observed as 0.9 and 1.2 fold in 250 μg/mL, 1.6 and 2.0 fold in 500 μg/mL, 2.8 and 4.4 fold in 1000 μg/mL and 3.4 and 5.6 in 1500 μg/mL concentrations of OSEO, respectively compared with OSEO untreated *F. graminearum* control (as expressed as 0 μg/mL in graphical representation **Figure 4C**).

Targeted genes encoding proteins were very significant in ZEA metabolism in synthesis of ZEA as well as release of ZEA to the media. This concluded that OSEO inhibited the production of ZEA by decreasing the mycelial biomass and also by downregulating the ZEA metabolic pathway genes (*PKS4* and *PKS13*). This could be explained by the effects of phenolic compounds on the structure and function of chromosomes. Significant reduction in the ZEA concentration at lower than MIC and MFC values of OSEO observed in the present study were clearly evidenced that, OSEO is more effective against ZEA production when compared with its antifungal activity. The results of the present study showed that, there was a clear lineage among mycelia biomass, target gene expression as well as ZEA production in liquid cultures.

Kumar et al. (2010) reported that OSEO was less toxic to mice (*Mus musculus* L.) compared to (LD_50_ value of OSEO as 4571.43 μg/kg) bio-preservatives like pyrethrum (350–500 mg/kg) and carvone (1640 mg/kg) (Isman, 2006). Extracts of *O. sanctum* L. possess immunomodulatory effects against *Salmonella typhimurium* infection in rat model by increase in TNF-α, IFN-**γ** and IL-2 cytokines generation (Goel et al., 2010) and also showed ameliorative effects on in sciatic nerve transection-induced neuropathy in rats (Muthuraman et al., 2008). In a review, Gupta et al. (2002) summarized various beneficial properties of *O. sanctum* L. and its use in ancient medicine including Ayurveda, Greek, Roman, Siddha, and Unani to treat several diseases. Keeping in view the importance of the *O. sanctum* in medicine and non-mammalian toxicity, its essential oil has the potential for use as a safe bio-fungicide of the agricultural commodities. Moreover, at 1500 μg/mL of OSEO concentration, the sudden fourfold decrease in mycelial biomass as well as insignificant ZEA production as recorded in present study is still uncertain. To understand the exact mechanism of OSEO on ZEA production at this concentrations, further studies are required to know the influence of OSEO on ZEA metabolic pathway regulation at genetic level.

### Application of OSEO onto Artificially Inoculated Maize Grains

**FIGURE 5:**
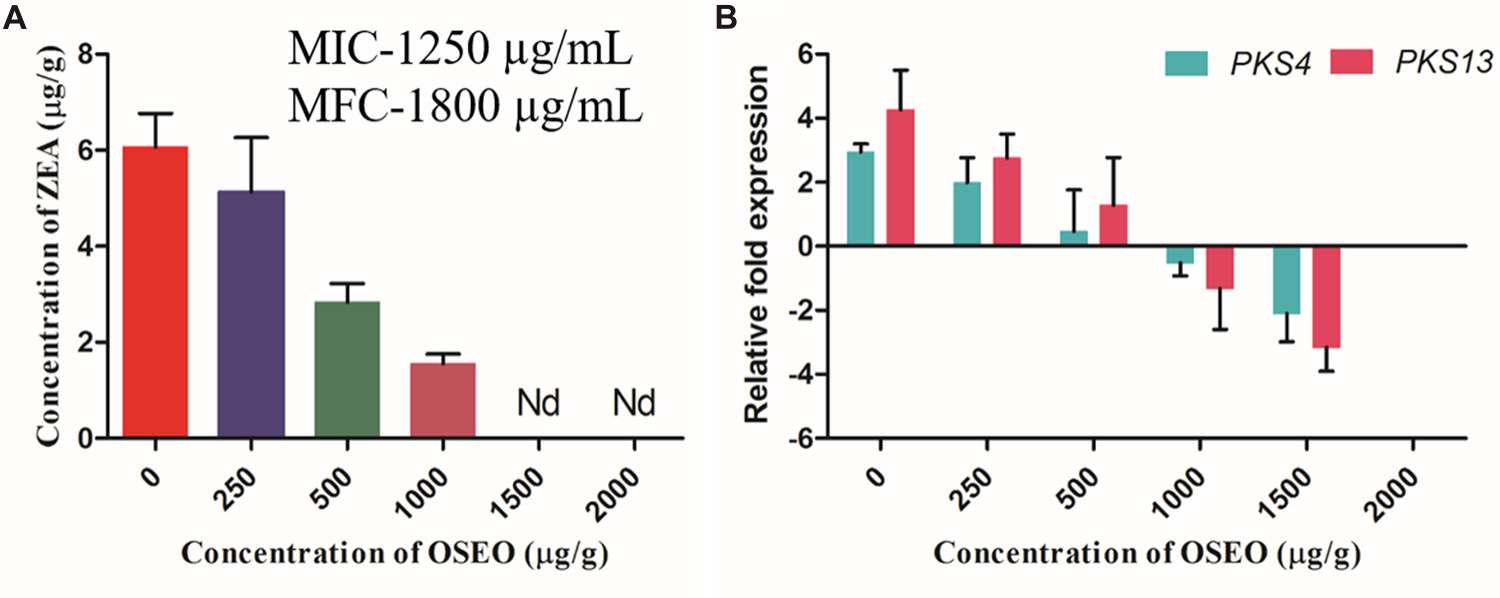
**Antimycotoxin activity of OSEO was performed onto maize inoculated with *F. graminearum* for ZEA production.** The experiment was conducted on maize grains at 28°C in a dark for period of 14 days. **(A)** Impact of OSEO on ZEA production quantified by reverse phase-UHPLC. **(B)** Impact of OSEO on zearalenone biosynthetic pathway genes expression (*PKS4* and *PKS13*) by Rt-qPCR. Data were analyzed applying a one-way ANOVA followed by the Tukey’s test (*n* = 6) and *p*-value <0.05. Nd, not detected.

To know the reliability and real time application of OSEO as an antagonist on growth and ZEA production of *F. graminearum*, studies on artificially contaminated maize grains were undertaken. Subsequently, UPHLC determination of ZEA revealed that, the decreased levels of ZEA in dose-dependent exposure of OSEO (**Figure 5A**). The concentration of ZEA production was significantly decreased with increasing in OSEO concentration viz, 5.12 μg/g in 250 μg/g, 2.83 μg/g in 500 μg/g and 1.55 μg/g in 1000 μg/g in comparison with untreated control (6.07 μg/g of ZEA). However, ZEA was not observed at a concentration of 1500 and 2000 μg/g of OSEO and the study clearly showed the inhibition of ZEA production significantly below MIC and MFC value of OSEO. The gene expression levels were downregulated upon treatment with OSEO to the samples, these results were in agreement with the liquid culture studies. The inhibitory effect of OSEO on target ZEA metabolic pathway genes *PKS4* and *PKS13* were depicted in (**Figure 5B**). A gradual decrease in the relative fold change in target gene (*PKS4* and *PKS13*) expression were observed upon increasing the concentration of OSEO. The observed fold change in target genes *PKS4* and *PKS13* were 1.0 and 1.5 fold in 250 μg/g, 2.5 and 3.0 fold in 500 μg/g, 3.5 and 5.6 fold in 1000 μg/g and 5.0 and 7.5 in 1500 μg/g concentrations of OSEO, respectively compared to OSEO untreated control. These results suggested that, upon increasing the concentration of OSEO, the expression levels of *PKS4* and *PKS13* genes were downregulated. The level of gene expression was low at the concentration of 1500 μg/g and no fungal growth was observed at the concentration of 2000 μg/g. The grain culture studies were well supported with the results of liquid culture analysis for ZEA production as well as target gene expression.

## Conclusion

In conclusion, by assessing the obtained results in the present study OSEO can be used as an herbal antagonistic agent against fungal infestation and ZEA production by *F. graminearum*. Owing the potential ill health effects of ZEA and *F. graminearum* on humans, animals and plant systems, the revelations of the present study indicates OSEO as an important intervention in food safety and processing industries where the fungal infestation is a main concern.

## Author Contributions

Design of the work; NK, VM, MS, BV, and VP. Interpretation of data for the work; NK, VM, GN, VG, and VP. Drafting the work; NK, VM, GN, VG, and VP. Final approval version to be published; NK, VM, and VP.

## Conflict of Interest Statement

The authors declare that the research was conducted in the absence of any commercial or financial relationships that could be construed as a potential conflict of interest.

## Abbreviations

OSEO: *Ocimum sanctum* L. essential oil
SDA: sabouraud dextrose agar
SDB: sabouraud dextrose broth
ZEA: zearalenone
